# A Solution to Reduce the Impact of Patients’ No-Show Behavior on Hospital Operating Costs: Artificial Intelligence-Based Appointment System

**DOI:** 10.3390/healthcare12212161

**Published:** 2024-10-30

**Authors:** Kerem Toker, Kadir Ataş, Alpaslan Mayadağlı, Zeynep Görmezoğlu, Ibrahim Tuncay, Rümeyza Kazancıoğlu

**Affiliations:** 1 Faculty of Health Sciences, Health Management, Bezmiâlem Vakıf University, Istanbul 34065, Türkiye; 2 Department of Information Technologies, Bezmiâlem Vakıf University, Istanbul 34093, Türkiye; kadir.atas@bezmialem.edu.tr; 3 Faculty of Medicine, Bezmiâlem Vakıf University, Istanbul 34093, Türkiye; amayadagli@bezmialem.edu.tr (A.M.); ituncay@bezmialem.edu.tr (I.T.); rkazancioglu@bezmialem.edu.tr (R.K.); 4 General Secretariat, Bezmiâlem Vakıf University, Istanbul 34093, Türkiye; zgormezoglu@bezmialem.edu.tr

**Keywords:** no-show behavior, hospital costs, artificial intelligence, hospital appointment systems

## Abstract

Background: Patient no-show behavior is a critical factor complicating hospital resource optimization and causing waste. The inefficiency caused by patients’ no-shows and the resulting increased operating costs negatively affect the hospitals’ financial structure and service quality. For this reason, health managers must make accurate predictions about whether patients will attend an appointment and plan the appointment system within the framework of these predictions. This research aims to optimize the hospital appointment system by making accurate predictions regarding the no-show behavior of the patients, based on recorded data. Methods: An artificial intelligence-based appointment system has been developed according to patients’ demographics and past behavior patterns. The forecast results and realized performance results were compared. The artificial intelligence we have developed continuously improves appointment assignments by learning from past and current data. Results: According to the findings, the artificial intelligence-based appointment system increased the rate of patients attending appointments by 10% per month. Likewise, the hospital capacity utilization rate increased by 6%. Conclusions: Findings from the study confirmed that no-show risks could be managed in the appointment process through artificial intelligence. This artificial intelligence-based design for appointment systems significantly decreases hospital costs and improves service quality performance.

## 1. Introduction

An aging population and rising healthcare spending have pressured providers to reduce costs and improve service quality [1]. Therefore, healthcare administrators must predict patient behavior to control operating costs and eliminate inefficiencies. One of the patient behaviors in question is not arriving for the appointment. Patients not using and canceling appointments are among the critical causes of inefficiencies in healthcare delivery [2]. Appointment systems allocate arrival dates and times to a particular facility or a group of facilities to serve clients [3]. It is a routine practice for hospitals to make an appointment in advance [1]. A hospital appointment system facilitates the exchange of information between the hospital and the patient [4]. No-show refers to patient behavior where the patient neither arrives for their appointment nor cancels it in advance [5,6]. This behavior jeopardizes the basic principles of primary healthcare, such as accessibility and the continuity of care [7]. This problem negatively affects service quality and dramatically increases service costs. Hence, this study aims to develop and test the accuracy of a model that predicts patients’ no-show behavior using artificial intelligence.

No-show rates vary between 3% and 80% in health systems, depending on the service provided and the demographic characteristics of the patients [8]. The average no-show rate reported in many previous studies of patient no-shows across various medical specialties is 23% [9]. In this regard, missing appointments causes problems that increase costs, reduce efficiency and productivity, and limit access to health services due to the inadequate use of appointment intervals and resources [10]. The monetary volume of these problems is large enough to merit discussion. For example, the no-show rate in the United Kingdom is around 12%, costing the health system GBP 600 million a year. Reducing this ratio from 12% to 10.8% will reduce the annual cost by one-tenth. In other words, an absolute reduction in the no-show rate of just 1.2% could save GBP 60 million annually [11]. Similarly, every missed hospital outpatient appointment costs NHS Scotland GBP 120, according to Scottish government data [12]. Also, a study by Kheirkhah et al. [13] found that the average cost of a no-show appointment was USD 196 per patient. Therefore, explaining no-show behavior and its determinants is crucial, as the practice wastes medical resources and jeopardizes the sustainability of healthcare services [4]. Hence, performance analysis of appointment systems is vital to determine current performance and to improve the system by evaluating different appointment scheduling alternatives [3]. Developing effective appointment solutions in line with these determinants is necessary.

An appointment scheduling problem typically involves minimizing the weighted cost of patient waiting, physician or clinic free time, and overtime [14]. In practice, hospitals and clinics have tried to implement various interventions to overcome the problem of no-shows (e.g., telehealth utilization, overbooking, and phone reminders) [15]. However, no-show issues persist, and significant benefits from these interventions are not realized [4]. Traditional approaches include overbooking and phone reminders, which have provided some short-term improvements but have not been effective in the long term. For example, overbooking is not always accurate and can result in long waits for some patients. Although telephone reminders provided a partial improvement in patient behavior, they did not reduce no-show rates to the desired level. In a health organization ecosystem where traditional approaches are insufficient in solving the no-show issue, a gap in the literature continues regarding original solution methods based on artificial intelligence.

In recent years, artificial intelligence-based prediction models have shown significant developments, especially in healthcare. Artificial intelligence has successfully predicted patient behavior by working on large datasets. Models such as regression analysis, decision trees, random forests, support vector machines, and neural networks have been used to predict patient no-show rates and have provided high accuracy. Xu et al. [16] identified factors such as service quality, excessive use, not knowing the details of the appointment, forgetting, waiting times, being late, intractable problems, time conflicts, and a lack of coordination as the reasons for no-shows. Huang and Hanauer [17] used a patient’s past no-show behavior as a predictor to investigate the effect on no-show predictability. According to the authors, the more a patient’s previous attendance/no-show status is considered, the better the prediction of a patient’s behavior toward the current appointment will be. Salazar et al. developed a prediction algorithm based on the random forest model to predict the no-show behavior of patients in Brazil. They predicted the patient’s no-show behavior and planned subsequent appointments based on parameters such as medical record number, gender, appointment date, scheduled appointment date attendance status, no-show reason, type of disability, date of birth, date of entry into the service, city, identifier of the patient’s disease, and which primary health unit referred the patient. The model could predict 92% of no-show patients accurately [5]. Artificial intelligence is a promising solution because it can analyze past data to predict the probability of each patient coming for an appointment, working in a personalized way, thereby providing more proactive solutions. In addition, with its continuous learning capacity, artificial intelligence can improve its performance by updating itself according to changing patient behavior.

Based on this reasoning, the main purpose of this study is to develop an artificial intelligence-supported prediction model to optimize appointment management in healthcare services. In this context, existing patient records were analyzed comprehensively, and the factors affecting the probability of appointments were determined. Using machine learning techniques such as decision trees, regression analysis, and information gain, the effects of variables such as the physician, branch, appointment time, and patient demographics on the probability of no-show appointments were examined. Based on the findings, an artificial intelligence algorithm was developed, automatically optimizing physician appointment planning by analyzing daily, monthly, and annual data. The developed model allows doctors to determine appointment slots as being either primary or secondary by estimating the probability of each appointment being kept. Thus, patient satisfaction increased, and the resources of the healthcare institution were used more effectively.

This paper is organized as follows: Section 1 critically examines the negative impacts of patient no-shows on healthcare efficiency and finances, exploring the existing solutions. Section 2 describes the materials and methods used throughout the study. Section 3 reports and explains the application’s results. Section 4 discusses the study by comparing the findings with the literature, and Section 5 presents the conclusion.

## 2. Materials and Methods

This study aims to design and evaluate an artificial intelligence-based appointment system (AI-BAS) capable of predicting patient no-show behavior. To achieve this goal, the following research methodology was employed.

### 2.1. Design

The study has been structured with an analytical approach based on data mining. In this context, we collected a large amount of patient and appointment data that was scattered across different systems on a single platform. We determined the critical factors affecting patient appointment behaviors by performing statistical analysis on this data using regression analysis and decision trees. These identified factors were used as input values in the AI-BAS we developed. Accordingly, the study consisted of four stages:

- The hospital appointment system and the factors affecting the effective operation of the appointment system were identified. Current literature and expert opinions were used to determine these factors.
- The patient characteristics determining the appointment no-show behavior were identified. Data analytics was used to determine these characteristics, and patient-specific behavioral models were created from the data patterns.
- Within the framework of the findings from the first and second stages, the AI-BAS model, based on actual data, was developed and tested in a university hospital and the application performance results were presented.
- The findings were discussed within the framework of the relevant literature.

### 2.2. Setting

Our study was conducted at a 600-bed university hospital in Istanbul, which receives approximately 5000 patient visits per day. The mean number of monthly appointments made via telephone and online is 78,000. ($\bar{x}$ = 78,361). Of these appointments, 60% are kept, 29% are canceled, and 11% result in no-shows.

### 2.3. Data Sources and Structures

The data were collected from the appointment system records between July and September 2022. These data consist of examination capacity, appointment cancellation rate, no-show patient rate, appointment realization probability, the number of appointments returning to the primary appointment from the substitute appointment, and the percentage of no-show patients. The demographic characteristics of patients and no-show behavior patterns were collected from the hospital database. Initial data analysis employed linear regression to categorize these features. This process identified significant predictors, which were subsequently incorporated into the algorithm. Outlier analysis revealed no anomalies. Additional data were integrated to enhance model accuracy, such as patients from the reserve pool being surveyed about their potential availability, and this information was used to prioritize appointments. The research was conducted under the ethical principles of the Declaration of Helsinki. To ensure patient privacy, personally identifiable information, including names, surnames, telephone numbers, and TR ID numbers, was excluded from the data-mining database. Before data collection, the study protocol received ethical approval from the Non-Interventional Research Ethics Committee of Bezmiâlem Vakıf University (Decision No: 2024/321).

**Figure 1:**
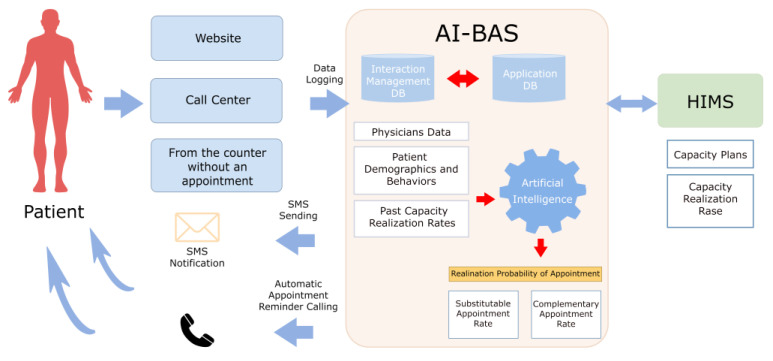
The operating logic of AI-BAS.

The AI system’s optimization parameters were derived from literature-based studies. These parameters are as follows: physician examination capacity, subject to monthly updates [14], appointment realization rate, cancellation rate, and no-show patient rate. These data can be divided into department-based, physician-based, and hour-based sub-breakdowns [15]. In terms of the probability of appointment realization, the algorithm analyzes the patient’s past behavior to identify these data. This analysis considers the patient’s previous cancellation behavior and instances of not attending appointments without cancellation. It also includes the patient’s age and patient address data in the modeling process [5]. The calculation conducted within the framework of these parameters attempts to produce the most effective estimate of the patient’s coming for the appointment. The user interface was constructed using a front-end framework, Bootstrap. On the back end, PHP version 8.1 served as the programming language, handling the server-side logic and data processing. A PostgreSQL database was utilized for data storage and retrieval. Figure 1 illustrates the operating logic of AI-BAS.

Figure 1 visually depicts the multi-step process of the AI-BAS, from data acquisition to analysis and decision-making. This framework will be analytically explained below.

### 2.4. System Architecture

In this phase, a conceptual model will be described that defines the structure and formalism of the AI-based appointment system. This plan describes the relationships between the different components of the system, how they communicate, and how, together, they form a whole.

#### 2.4.1. Data Layer

- Patients’ appointment history: Appointment date, appointment time, physician, branch, and appointment status (completed, canceled, and no-show).
- Patients’ demographic information: Age, gender, and address.

#### 2.4.2. Data Processing Layer

- Data cleaning and pre-processing: Correcting missing or inconsistent data and making it ready for processing.
- Feature engineering: Creating new features from data that will improve the performance of the prediction model.
- Data normalization: Normalizing the data to prevent it from being at different scales.

#### 2.4.3. Data Science

- Appointment Cancellation and No-Show Prediction Model: This model predicts the probability of patients canceling or not showing up for appointments, determines the number of reserve patients accordingly, and opens this pool to reservations.
- Model training: Teaching daily, monthly, and annual data to the instant prediction algorithm.
- Model selection: Extracting the features of canceled and no-show appointments using regression analysis and decision tree methods. Regression analysis is convenient for estimating no-show probabilities by modeling the relationships between dependent and independent variables. Common socio-demographic and clinical characteristics may be shared between patient groups. This increases the relatability among patients who have appointments with the same healthcare provider. For example, patients in the same clinic may exhibit similar no-show behaviors because these patients usually have similar socioeconomic conditions and demographic characteristics. Naturally, this clustered structure should be considered in statistical analysis. Ignoring such dependencies may lead to biased variance estimation and erroneous results. Therefore, it is desirable to consider the semi-parametric regression analysis of clustered panel count data [18]. Decision trees are supervised learning models constructing a series of if-else questions to classify or predict outcomes based on input features. The decision tree model was chosen for its high interpretability and ability to handle complex, nonlinear interactions between variables such as demographics and past appointment behaviors. This method has proven effective in healthcare prediction tasks because it provides clear decision paths that make it easy for healthcare professionals to understand the logic behind each prediction. Also, the decision tree strikes a fundamental balance between accuracy and interoperability. This balance is crucial in environments where actionable insights are needed in real time to manage appointment scheduling efficiently. These two methods are based on the information gain principle, which improves the model’s performance and obtains the best results. Information gain is a metric often used in machine learning algorithms, especially decision trees. It measures how much new information is provided when a dataset is split based on a specific feature. In other words, it is a quantitative indicator of how well a feature can predict the target variable. This calculation starts by measuring the uncertainty (entropy) in the dataset. Entropy indicates how homogeneous the distribution of classes is in the dataset. Then, the dataset is split into subsets according to the selected feature, and the entropy of each subgroup is calculated. Information gain is obtained by subtracting the weighted average entropy of the subsets from the initial total entropy. This difference expresses the success of the selected feature in distinguishing classes in the dataset. For example, if patient age significantly predicts no-show behavior, the decision tree will split on this feature, leading to more homogeneous subgroups with higher prediction accuracy.

#### 2.4.4. Decision-Making Layer

- Determining the number of backup appointments needed for canceled appointments and patients unable to attend: The estimation algorithm’s results determine the number of backup appointments, which are automatically assigned through the website where patients apply for appointments.
- Creating a complementary appointment: The estimation model is instrumental in this step, as it guides the creation of complementary appointments to minimize the possibility of cancellations or no-shows.
- Optimizing appointment planning: Optimizing appointment planning by considering current and complementary appointments. Accordingly, appointment slot quotas in the relevant branches are determined.

Using the PHP programming language, algorithms have been developed using loops, arrays, and functions that perform specific functions. These algorithms are designed to complete various tasks, such as data processing and model execution. The cronjob is a timer that automatically performs those tasks that must be run at specific intervals or periodically. These tasks manage instant data retrieval and processing processes from various systems. Data on the physicians’ working hours and appointment schedules are retrieved via the hospital information management system (HIMS), then patient appointment requests are received via the appointment management system, and automatic callback results concerning whether patients will come to the hospital are collected via the IP switchboard system. These algorithms, written in PHP, are integrated with cronjob tasks, enabling instant retrieval, processing, and inclusion of data from these different sources into the system at specific intervals. Thus, the data in the appointment system are dynamically updated, and the prediction performance is improved by continuously providing new data to the artificial intelligence model. The detailed parameters we used in the design of the artificial intelligence algorithm and their definitions are as follows:

(1) Examination capacity (EC): This indicates the number of monthly examination appointments determined by the department of each hospital physician. The number of appointments is calculated as follows: physicians declare their average appointment times, and this statement may be updated annually. In contrast, department secretaries collect monthly work schedules from the physicians. This work schedule is transferred to the HIMS. Then, the HIMS automatically opens the number of daily appointments according to the work schedule from the average examination period.

(2) Appointment realization probability (ARP): This shows the ratio of the number of appointments made online or through the call center to the total number of appointments.

(3) Appointment cancellation rate (ACR): This shows the ratio of the number of cancellations made by patients who make appointments online or through the call center to the total number of appointments.

(4) No-show patient rate (NSPR): This shows the ratio of the number of no-shows by patients who made an appointment online or through the call center to the total number of appointments.

(5) Primary appointment (PA): The primary examination appointment is based on the physicians’ average examination times and calendars.

(6) Substitute appointments (SA): The appointment given to another patient after the appointment became available after a no-show, allocated after the work schedule determined for the relevant physician is complete, is called a substitute appointment. An alternate appointment is one made when the system assigns it as the primary appointment instead of the canceled appointment. In addition, substitute appointments are also assigned as complementary appointments, as predicted by the artificial intelligence algorithm.

(7) Complementary appointments (CA): This refers to appointments scheduled based on the estimated number of no-show patients and the level of cancellations, calculated to ensure there are enough spare appointments available.

The optimal sequencing problem arises when patients are classified according to specific characteristics, such as service time distribution, the average length of service, variability in service, the probability of no-show, and the unit cost of waiting [3]. Therefore, classifying patients according to their behavioral and demographic characteristics is critical in predicting no-show behavior. Creating the most appropriate no-show and walk-in scenarios for fit patient clusters is essential to achieve optimal system performance. The system’s performance is measured by how well it manages doctors’ appointments. The AI aims for full occupancy, but physicians may face difficulties in adjusting to more patients. The system adjusts the AI’s recommendations to help physicians gradually reach 100% occupancy. For this purpose, the formula for calculating the number of substitute appointments and the rule for conversion to the primary appointment represent the basis of the AI-BAS algorithm.

The number of substitute appointments, SA, is calculated as follows:

SA = (EC × ACR) + (EC × NSPR)(1)

where EC represents examination capacity, and ACR and NSPR denote the appointment cancellation and no-show patient rates, respectively.

The rule of conversion of the substitute appointment to the primary appointment is determined so that when the primary appointment is canceled, the first appointment taken as a substitute becomes the primary appointment. The relevant patient registered for a substitute appointment is informed via SMS.

The complementary appointments, CA calculation rule is given as follows:

CA = [(EC × ACR) − (ARP × AR)] + EC × NSPR(2)

Here, ARP, AR, and NSPR represent the appointment realization probability, the number of appointments returning to a primary appointment after being a substitute appointment, and the percentage of no-show patients, respectively.

#### 2.4.5. Presentation Layer

- User interface: A website appointment management screen has been developed to allow hospitals and patients to use the system and manage appointments. In addition, the system operates entirely in the background and is only accountable to the chief physician and information technology team. The system analyzes the data of three hospital systems (the HIMS, appointment website, and callback reminder search system), and the estimation algorithm works automatically and does not allow manual intervention. Therefore, the system is managed by a single administrator.

#### 2.4.6. Integration Layer

- Hospital information system integration: We have established direct integration with the HIMS application and website, ensuring seamless compatibility. The web service and the instantaneous movements of the data are monitored by the estimation algorithm.

**Figure 2:**
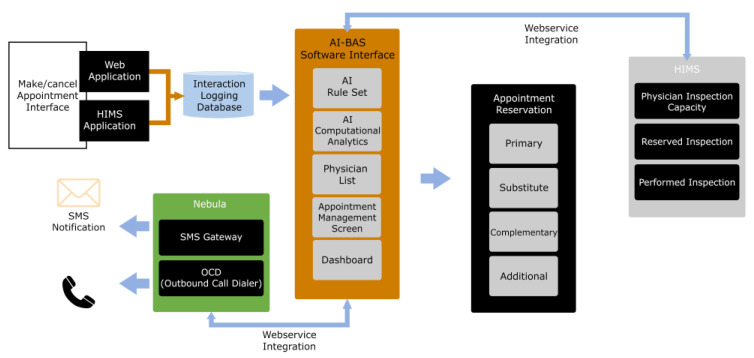
The system architecture of AI-BAS.

Figure 2 shows the system architecture of AI-BAS and the interactions between the components.

The AI algorithm used in the study consists of regression analysis and decision tree methods. Regression analysis predicts patient behavior by modeling the relationships between dependent and independent variables. This technique analyzes the connections between patient demographics, past appointment behaviors, and geographic data. In this way, the probability of a patient coming to an appointment can be predicted with high accuracy. Conversely, decision trees perform classification operations by dividing datasets into smaller groups and detecting distinct patterns in the data structure. The decision tree model determines which patients are more likely to miss an appointment by performing a layered analysis of patient data. The information gain metric determines what features (e.g., age, gender, and past appointment status) would increase the model’s predictive accuracy the most. This is a critical step in strengthening the model’s predictive ability. The criteria used in selecting AI algorithms are primarily based on data accuracy, the model’s ability to adapt quickly, and its ability to provide consistent results with large datasets. Decision trees exhibit remarkable adaptability, allowing for the seamless integration of new data and dynamic adaptation to evolving patient trends. When confronted with extensive datasets, these algorithms consistently deliver reliable results, making them well-suited for the demands of modern hospital operations. While alternative algorithms like k-nearest neighbor and support vector machines may offer comparable levels of accuracy, they often introduce complexities in terms of interpretability and operational implementation. Decision trees emerge as a preferred choice in healthcare services, where transparency and interpretability are paramount, due to their inherent understandability.

The PHP Version 7.4.28 programming language has been used to create software infrastructure that enables the execution of these algorithms. Mathematical modeling alone is not enough to turn artificial intelligence algorithms into solutions; software tools are used to allow these models to work with real-time data and make dynamic predictions. In this study, prediction functions have been developed with the PHP language, and the data processing processes running in the system’s background are carried out using this software language. PHP is preferred for the software development process because it is a language widely used for dynamic web applications and has broad community support. PHP offers rapid development opportunities, especially in web-based projects, and manages server-side operations efficiently. Since this study aims to develop a system with a high user-interactive interface, using PHP was deemed more appropriate. In addition, considering the experience of the existing software development team in PHP, the project progressed quickly and smoothly. The PostgreSQL database was preferred in terms of data reliability and scalability. Its performance, especially in processing high-volume data, along with its ACID (atomicity, consistency, isolation, and durability) compliance supported this choice for securing critical data, such as hospital data.

### 2.5. Plot Study

AI-BAS was practiced as a pilot study in a limited number of outpatient clinics (2 clinics) in July 2022. During this phase, based on last year’s data, we calculated the probability of making an appointment, based on physician and branch. Here, the algorithm analyzed patient cancellation habits for each physician and unit and produced a probability score for a newly entered appointment. This score provided updates accordingly as data from each new day came in. After the redefined rules, procedures, and policies were tested in the second pilot study (20 clinics), the identified shortcomings of AI-BAS were improved. In this phase, we analyzed cancellation behaviors according to appointment time. Cancellations are a critical issue among all the uncertain factors. Patients often cancel their appointments just before their scheduled time, leading to last-minute cancellations. If patients cancel their appointments far enough in advance, the hospital can allocate the vacant position to other patients [19]. We looked at the cancellation rates and added this parameter to our algorithm. We analyzed the cancellation behaviors according to the patient’s location (province and district) and added the location parameter to our algorithm. In addition, we scored patient cancellation behaviors according to age range. We classified the patients as 0–10, 11–20, 21–30, 31–40, 41–50, 51–60, and 61–70 years old. We found that the probability of appointment cancellation increased with increasing age. We analyzed the cancellation behavior by gender and added it as a parameter to our algorithm. We also asked patients for data on how long it would take them to arrive at the hospital. Accordingly, we prioritized converting spare patients to primary appointments and then ranked the extra patients. With this strategy, the appointment realization rates significantly increased. The appointment realization rate nearly doubled compared to the pilot application period in July and August. The system was put into operation at total capacity in August 2022.

## 3. Results

**Figure 3:**
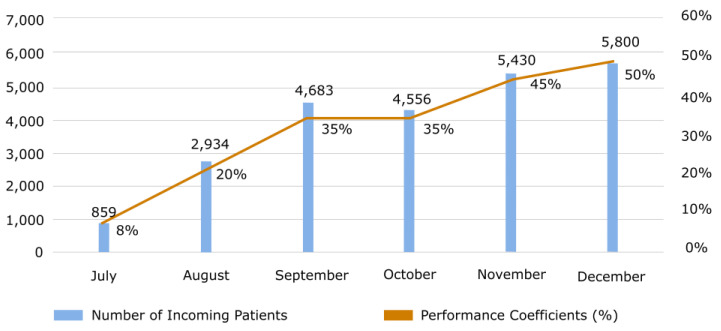
AI-BAS performance coefficients and the number of incoming patients.

In July 2022, when the pilot study was conducted, the number of walk-in patients increased by 859 via the appointment scheduling realized by AI. However, after improving the system by addressing the issues identified during the pilot study, the appointment system became operational for all hospital services in August. We have achieved the crucial outcomes. The increase in the number of patients who could be examined in August was 2934, 4683 in September, and 5800 in December. AI-BAS pre-detected the patient cluster with no-show potential by correctly analyzing and interpreting the appointment data. The system made replacement patient assignments based on the data pattern of this identified patient cluster. Thus, increased productivity was achieved by reducing the idle working capacity of physicians. Statistically, the mean of five-month patients coming through AI-BAS was 4680, the standard deviation was ± 1104, and the median was 4683. Figure 3 shows the change in the number of substitute patients arriving at the hospital instead of the no-show patients recorded monthly.

Figure 3 also shows the monthly performance progress and its positive tendency. System performance measurement is based on a parameter representing the number of patients who previously demonstrated no-show behavior. We achieved an 8% performance improvement in the pilot study when we transitioned to AI-BAS. A 20% performance increase in August was followed by a 35% increase in September. Given the data’s non-normal distribution, a Spearman rank correlation coefficient was employed to assess the relationship between variables. The system performance reached 50% at the end of December. A Spearman correlation analysis revealed a strong, positive association (r = 0.95, *p* < 0.01) between system performance and the number of patients from AI-BAS. Examining the differences between predicted and actual performance in detail is a critical step toward improving the system. The differences observed between our study’s expected and actual results may be due to changes in patient behavior, especially during specific periods. For example, external factors such as appointment times, the regions where the patients live, or seasonal changes may have affected the no-show rates and caused differences between our predicted and actual performance. When analyzing these deviations considering the performance increase observed in the pilot studies, especially in the first months of the system, it was predicted that the model’s learning capacity would increase over time and produce more accurate predictions. In particular, the 8% performance increase, reaching 35% quickly, clearly shows an improvement in the model’s accuracy. In future studies, we plan to enrich our dataset with finer details and better model the external factors to understand the reasons for these deviations better.

**Figure 4:**
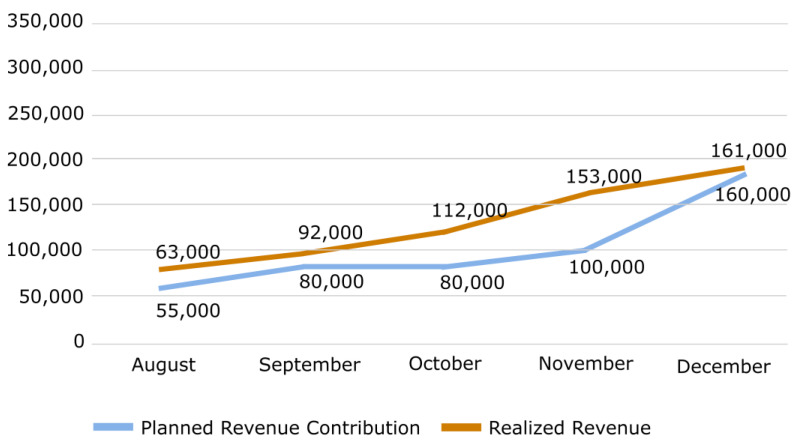
AI-BAS planned–realized monthly examination revenue impact in USD.

By detecting patients in advance and creating an effective patient appointment substitution pattern, AI-BAS no-show identification increased the total system performance and financial performance. In July (the pilot study), AI-BAS generated USD 20,610 in income for the hospital. In the following months, revenue dramatically increased with implementing the implementation of the system across all health services. The contribution to total revenue was nearly USD 63,000 in August and over USD 92,000 million in September. The impact of AI-BAS on monthly income was USD 161,000 at the end of December. Figure 4 shows the positive effect of AI-BAS on hospital income.

Concordantly, AI-BAS increased the realization rate of physicians’ examination capacity plans to boost productivity. Canceled and vacant appointments left by patients who were no-shows were causing the monthly capacity utilization rate to be between 75% and 85%. Spearman correlation analysis showed a statistically significant 0.975 relationship between the inspection income that we planned through AI-BAS and the actual income (*p* < 0.01). The Spearman correlation analysis results show that the AI-BAS model’s predictions are highly consistent with reality. This proves the model’s reliability and highlights the importance of healthcare institutions making data-based decisions. Table 1 shows the improvement that AI-BAS provided in the hospital performance indicators.

Owing to the transformation of AI-BAS substitute appointments into primary and actual appointments, the capacity utilization rate increased by around 6%. The rate of patients with appointments increased by about 10% on average. We served more than 5500 patients per month. Also, AI-BAS reduced the physicians’ administrative workload, allowing them to spend more time directly on patient care.

## 4. Discussion

Artificial intelligence-based appointment systems in healthcare are rapidly developing, and contributions in this area are diversifying daily. While AI models in the literature generally focus on predictive capabilities, our study stands out in terms of integrating these predictions into real-time decision-making processes. For example, many AI models only predict the probability of patients not coming for an appointment with past data, and these predictions are implemented in a static system. However, our model integrates the output of the artificial intelligence algorithm directly with operational decisions with the dynamic appointment management system. This aligns with the proactive decision support systems approach, which is increasingly gaining importance in AI literature. Studies such as that by Hekler et al. [20] indicate that predictions should be integrated with real-time decisions for prediction models to be effective in healthcare. In our model, this integration allows for filling empty appointments by assigning substitute patients instead of patients who present a high risk of not attending an appointment and optimizes the use of hospital resources. In addition, the principle of continuous learning, which is increasingly accepted in the literature, is a critical part of our model. Many existing AI models remain static after being trained with the initial data and cannot adapt to changing patient behaviors over time. However, our model increases its predictive capabilities by constantly updating itself with new data and responding more effectively to possible future behavioral changes. This is directly related to the approaches known as continuous learning and incremental learning, as found in the AI literature [21]. In this context, our algorithm exhibited a 1% increase in accuracy over random forest algorithms, leading to more precise predictions and potentially improved decision-making. It offers an innovative solution by integrating the increasingly important proactive operational decision-making and continuous learning approaches in the literature. In this respect, our study contributes to existing AI models.

The system we have developed gives patients precise reminders about their appointments and appointment-booking instructions, increasing their likelihood of attending them. Yet, in the context of pre-service appointment management, handling client absenteeism poses a significant challenge [11]. Research on appointment scheduling in the literature consists of two principal methodologies: analytical and simulation studies. Analytical studies use queuing theory, mathematical programming, and dynamic programming to determine the number of patients or the interval length for each appointment interval (slot, session, or day). In contrast, simulation studies focus on specific appointment scheduling systems in complex environments to compare the various systems and measure the impact of critical factors [22]. Which technique will be used in appointment planning depends on the organization’s transaction volume, its complexity, and the uncertainty of patient behavior. Due to our extensive and detailed data capacity, we preferred to use the analytical approach first and then let the artificial intelligence learn. Thus, our AI algorithm has created a dynamic prediction model using data analysis and machine learning techniques. The flexible structure of AI-BAS is provided by its ability to respond instantly to the patient’s circumstances and by its ability to predict possible future cancellations by analyzing patient behavior. The system increases the flexibility of the appointment system and maximizes hospital efficiency by producing more accurate predictions over time with its continuous learning mechanism. This model predicts future behaviors using multidimensional data such as the patient’s demographic information (age, gender, and address) and past appointment behaviors (previous appointment attendance status, cancellations, etc.). Our AI system processes instantly updated data to increase appointment occupancy and provides the most appropriate patient–physician match. In this way, the hospital’s resources are used more efficiently, the time that physicians spend idle is reduced, and more patients are served. AI-BAS’s real-time data processing capacity effectively manages last-minute changes, considerably contributing to overall system performance.

Previous studies on health information systems and their management have generally agreed that patients’ absenteeism is not arbitrary; they have investigated its determinants, which include gender, age, referral source, appointment time, transportation services, and weather conditions [4]. Reasonable estimates of participation levels provide insight into improving policy assessment, minimizing the undesirable effects of resource allocation practices (such as overbooking), and identifying influential stakeholders [23]. According to Huang and Zuniga [9], solutions have been proposed in the literature to reduce the number of missed appointments and the adverse effects of broken appointments, but the problem persists. In the context of the literature, we reviewed the substitute appointment parameter and complementary appointments that we use in our algorithm, which effectively addresses no-show problems. AI-BAS determines which patients in the pending portfolio will receive backup appointments. Inviting the substitute patient who is most likely to arrive at the hospital actively eliminates system gaps.

Moreover, practitioners should consider the reservation horizon when designing their appointment system. The reservation horizon determines how far in advance an appointment can be scheduled and is an input parameter to the appointment system [2]. Also, the appointment age refers to how long ago an appointment was made. As appointment age increases, the likelihood of patients exhibiting no-show behavior also increases [24]. As another appointment rule, the dome rule is a variable-interval, single-block rule, with appointment intervals gradually increasing toward the middle of the session and then gradually decreasing toward the end [13]. All the mentioned appointment system rules aim to minimize the difference between planned and actual capacity. AI-BAS has also solved the reservation horizon problem. Thus, based on the data derived from patient location, appointments can be made for patients who can come to the hospital on that same day. The system can call on patients up to one hour before the appointment. Thus, AI-BAS, in contrast, has eliminated the gaps in the appointment system and increased efficiency by assigning appropriate substitute patients, in terms of time and their location, instead of the primary patients.

Researchers have recommended different solutions to address no-shows in the literature. For example, Cao et al. found that since the implementation of an internet-based hospital appointment system, patients’ waiting times for registration have decreased from 98 min to 7 min [25]. Jerbi and Kamoun [26] found, in their study in a hospital in Tunisia, that the best schedule to use is an appointment system with the “new patients first” scheduling rule and the “individual block” appointment rule. Unlike these rules, AI-BAS determined those patients with the highest potential to exhibit no-show behavior from the existing patient appointment portfolio and then created substitute appointments. In this way, our algorithm has developed an understanding of solving the patient-flow issue before the patients arrive at the hospital. According to Woicik et al. [27], if the first appointment is missed, the probability of the patient not attending at a later date is ten times higher. In line with this insight, it was deemed necessary to accurately determine the likelihood of appointment loyalty to manage the risk of no-shows. Therefore, our study examined patients’ past appointment commitment behavior and analyzed the relationships between this behavior and demographic characteristics. The results obtained in our study show a significant improvement compared to similar AI-supported appointment systems in the existing literature. For example, the 50% increase in system performance by December is a significant improvement compared to previous studies [5,13,16,17]. In the literature, performance improvements of between 20% and 35% have generally been reported in similar AI appointment systems. In this context, the 50% improvement offered by the AI-BAS model exceeds the industry’s current success criteria, reinforcing the model’s effectiveness and application potential. Similarly, with the developed AI system, the hospital’s capacity utilization rate increased by 6%, appointment attendance rose by 10% monthly, and the appointment system’s efficiency significantly boosted financial productivity. Accordingly, we generated an extra income of approximately USD 570,000 via AI-BAS in the first five months. This gain achieved through AI-BAS is not limited to serving more patients but has also achieved the more efficient use of hospital resources (workforce, facilities, and time). According to projections, the USD 1.7 million increase in revenue expected to be achieved with its full implementation is directly related to optimizing the appointment system. This improvement has allowed for more flexible and effective use of hospital resources, increased patient satisfaction, and an increased likelihood of repeat service.

Appointment systems can be a source of dissatisfaction for both patients and providers. Patients are adversely affected by the lack of timely and convenient appointment intervals, especially when their needs are urgent. Clinicians are affected by uncertainty regarding the number of daily patient appointments and the mix of appointments on any given day [28]. An effectively designed appointment system should strike a balance between the efficiency of service providers and the satisfaction of patients. However, many patients do not attend appointments, even though the physicians schedule an entire workday [1]. According to Jerbi and Kamoun [25], environmental factors such as non-attendance may also affect the appointment system, timing, and rules. For this reason, conducting a comprehensive root cause analysis for business and location-based no-show behavior and designing a tailor-made appointment system based on these reasons will increase system performance. In this study, a dynamic callback and patient information system that is integrated with our AI-based algorithm has been developed to minimize and optimize no-show behavior. This system ensures that patients with appointments are called at a specific time before the appointment date to confirm their attendance status. This verification process aims to proactively detect situations where patients are considering canceling their appointments or are unlikely to attend. The AI algorithm analyzes the patients’ previous appointment behaviors, identifies patients with a high probability of no-show, and creates a risk profile for these patients. The algorithm includes the patient’s age, gender, address, and past appointment attendance factors when making this profile. These high-risk patients are then called via the system’s callback software VoiceXML 3.0, and CCXML 1.0 Version, and their attendance status is confirmed. If the patient reports that they cannot attend their appointment, the system automatically cancels this appointment slot and directs them to the reserve patient pool. The reserve patient pool is optimized according to the no-show rates predetermined by the AI algorithm. Reserve patients are informed via SMS messages or automatic announcement calls and invited to come to the hospital for an appointment. These SMS reminders prevent patients from forgetting their appointments and encourage them to arrive on time [29]. This process is repeated continuously to minimize gaps in the hospital appointment system and to utilize the doctors’ time most efficiently. The AI algorithm can continue this process until the specified number of reserve patients arrive, minimizing the risk of empty appointment slots. Thus, the AI algorithm reduces the no-show rate and ensures the efficient use of hospital resources.

Our research findings revealed the positive contribution made by an AI-supported design and implementation of the hospital appointment system to operational and managerial performance outcomes. In particular, we emphasize the importance of past appointment data and patient demographics in predicting a patient’s no-show or walk-in behavior. Also, even the most sensitive planning in hospital operation management processes carries uncertainties. Uncertainties may arise primarily from the randomness between scheduled and unscheduled patients’ arrival times, no-shows among expected patients, randomness in the length of service at various stages of care, uncertainties in a patient’s health status transitions, and transitions between different care locations [30]. Therefore, data structuring to reduce patient-induced uncertainties is critical in the design of such artificial intelligence algorithms. This matter requires the practical application of a turn-based appointment system in outpatient planning, categorizing patients into manageable groups and assigning them to pre-marked clusters when called for an appointment [24]. For this purpose, we classified the data expressing appointment realization, cancellation, and no-show rates, based on branches, physicians, and hours. This classification was a reasonable attempt to predict uncertainties in patient behavior. In this regard, more patients could receive health services via AI-BAS. According to Yang et al., [31], with an appropriate appointment system, hospitals establish better relationships with patients and provide care to more patients. AI-BAS enabled patients in need of treatment to visit the hospital by making a substitute appointment. Also, there are two beneficial aspects regarding patient satisfaction. Firstly, we ensured patient satisfaction by providing health services to patients needing treatment. Secondly, we prevented the formation of a queue by ensuring that the additional patients visited the hospital using a planned approach. AI-BAS enabled patients without an appointment to receive health services after a reduced waiting time by registering them as substitutes instead of the patient having to wait all day. Therefore, reducing the uncertainties has helped avoid potential physician–patient conflicts.

Appointment scheduling systems are at the intersection of efficiency and timely access to healthcare. Convenient access to healthcare is essential for achieving satisfactory medical outcomes. It is also vital to patient satisfaction [22]. Therefore, if the appointment system is not designed well, it can be a source of dissatisfaction for both patients and healthcare professionals [26]. This issue may cause the patient not to choose the hospital in question again, and it may also cause the healthcare professionals to leave for jobs in other institutions. In this context, AI-BAS makes a direct financial contribution to hospital management processes and offers performance effects such as customer service development and human resource satisfaction. Küçük et al. [32] stated that patient flow and capacity planning are essential factors for improving the performance of an outpatient department. Our operational results, summarized in Table 1, support this argument.

Organizations must ensure customer satisfaction and create value while minimizing total costs [33]. Therefore, system performance is a critical parameter that determines the effectiveness of hospital appointment systems. To achieve positive capacity utilization, organizational/managerial improvements such as increased patient satisfaction and employee satisfaction are also required. According to Safdar et al. [34], a short patient waiting time is one of the critical criteria when evaluating patient satisfaction, service quality, efficiency, and capacity planning. In all the critical indicators we explained above, AI-BAS contributed to increased hospital operation efficiency and reduced costs. Also, the AI-BAS algorithm improves system optimization by constantly learning from past and current appointment data patterns. Our algorithm has saved our hospital an extra day each month without investing any additional resources, simply by changing our perspective on the appointment system.

Although our study was conducted at a single hospital, recognizing this can enhance our model’s generalizability. Thanks to its big data analytics and artificial intelligence techniques, it is designed to be flexible enough to adapt to different datasets. Also, the scalability of the AI-BAS model varies, depending on the variety and amount of data in various hospitals. While large datasets from large hospitals increase the model’s performance, the limited data available in small hospitals raises questions about the model’s effectiveness. However, with the model’s flexible structure, adaptations can be made for small hospitals or clinics with less data. Our pilot applications provide a vital reference for the model’s applicability to other hospitals.

### Limitations and Recommendations for Future Research

Our study has several limitations. Firstly, our algorithm was adapted for use in a single hospital. Adapting the algorithm to other healthcare organizations may require the consideration of different parameters, such as changes in average examination times and seasonal fluctuations. Secondly, while our analysis provides valuable insights, it is limited by excluding seasonal patterns, insurance status, and sociocultural differences. Future research strategies should address these gaps by incorporating these factors into the analysis and testing the algorithm’s cross-cultural applicability. Finally, although the dataset was obtained from existing hospital data, training and test sets were not created, and standard evaluation metrics and cross-validation techniques were not applied during model training. This situation limited our opportunity to evaluate the model’s performance more systematically. However, the study was conducted to understand and improve the essential operation of an AI-supported appointment system, and the findings thus obtained are valuable for the initial phase. In future studies, applying these methods to increase the model’s reliability and test its performance with a more extensive dataset is recommended.

## 5. Conclusions

In this study, an artificial intelligence-based appointment system, developed by analyzing past patient data and demographic characteristics, is used to manage appointment cancellations by identifying patients with a high likelihood of no-show behavior, assigning backup appointments, and offering alternative times to patients. In this way, the system reduces the number of patients waiting to access healthcare services and ensures the more efficient use of existing resources. While the algorithm improves doctors’ time management, it also increases the institution’s capacity and allows it to serve more patients. Thus, patient satisfaction increases and costs are reduced, optimizing the hospital revenues. The algorithm’s prediction accuracy can be further increased in future studies by adding different social, economic, and psychological parameters.

## Figures and Tables

**Table 1:** Changes in appointment parameters, shown according to month.

Months	June (Before AI-BAS)		July (Pilot)		August		September		October		November		December	
Parameters	*n*	(%)	*n*	(%)	*n*	(%)	*n*	(%)	*n*	(%)	*n*	(%)	*n*	(%)
The number of Web Appoint.	92,527	100	62,110	100	77,389	100	78,696	100	75,324	100	81,966	100	80,481	100
Realized Web Appoint.	52,846	57.1	36,008	58.0	44,307	57.3	45,789	58.2	43,513	57.8	46,748	57.0	45,170	56.1
Web Cancel Appoint.	29,227	31.6	18,605	30.0	23,341	30.2	23,043	29.3	22,544	29.9	26,321	32.1	25,797	32.1
Web No-Show	10,454	11.3	7497	12.1	9741	12.6	9864	12.5	9267	12.3	8897	10.9	9514	11.8
Realized AI-BAS			859	1.3	2934	3.7	4683	6.0	4556	6.1	5430	6.59	5800	7.3
Realized Web + AI-BAS			36,867	59.2	47,241	61	50,472	64.2	48,069	63.8	52,178	63.6	50,970	63.4
The ratio of AI-BAS-Realization to Web Realization				2.2		6.5		10.4		10.5		11.6		12.9

## Data Availability

The dataset generated during the current study is not publicly available. The study dataset is available from the corresponding author upon reasonable request.
